# A Robust Gradient Based Method for Building Extraction from LiDAR and Photogrammetric Imagery

**DOI:** 10.3390/s16071110

**Published:** 2016-07-19

**Authors:** Fasahat Ullah Siddiqui, Shyh Wei Teng, Mohammad Awrangjeb, Guojun Lu

**Affiliations:** 1 Faculty of Information Technology, Monash University, Clayton VIC 3800, Australia; 2 Faculty of Science and Technology, Federation University Australia, Churchill VIC 3842, Australia; shyh.wei.teng@federation.edu.au (S.W.T.); guojun.lu@federation.edu.au (G.L.); 3 School of Information and Communication Technology, Griffith University, Nathan QLD 4111, Australia; m.awrangjeb@griffith.edu.au

**Keywords:** building extraction, LiDAR, photogrammetric imagery, transparent roof building, dense vegetation, small size building

## Abstract

Existing automatic building extraction methods are not effective in extracting buildings which are small in size and have transparent roofs. The application of large area threshold prohibits detection of small buildings and the use of ground points in generating the building mask prevents detection of transparent buildings. In addition, the existing methods use numerous parameters to extract buildings in complex environments, e.g., hilly area and high vegetation. However, the empirical tuning of large number of parameters reduces the robustness of building extraction methods. This paper proposes a novel Gradient-based Building Extraction (GBE) method to address these limitations. The proposed method transforms the Light Detection And Ranging (LiDAR) height information into intensity image without interpolation of point heights and then analyses the gradient information in the image. Generally, building roof planes have a constant height change along the slope of a roof plane whereas trees have a random height change. With such an analysis, buildings of a greater range of sizes with a transparent or opaque roof can be extracted. In addition, a local colour matching approach is introduced as a post-processing stage to eliminate trees. This stage of our proposed method does not require any manual setting and all parameters are set automatically from the data. The other post processing stages including variance, point density and shadow elimination are also applied to verify the extracted buildings, where comparatively fewer empirically set parameters are used. The performance of the proposed GBE method is evaluated on two benchmark data sets by using the object and pixel based metrics (completeness, correctness and quality). Our experimental results show the effectiveness of the proposed method in eliminating trees, extracting buildings of all sizes, and extracting buildings with and without transparent roof. When compared with current state-of-the-art building extraction methods, the proposed method outperforms the existing methods in various evaluation metrics.

## 1. Introduction

Building extraction from remotely sensed data is being used for many different applications, e.g., disaster monitoring, real estate, national security and public service centres for solving the human welfare matters, especially at the time of an accident, disaster or combat situation. However, the automatic extraction of buildings from remotely sensed data is one of the challenging tasks faced by the computer vision and remote sensing communities. This task is challenging due to many reasons such as complexity in the building structures, surrounding environment (highly-dense vegetation, occluded building and hilly scene), poor acquisition of data and registration error between data sources.

There are mainly two different sources of input data, i.e., the photogrammetric imagery and Light Detection And Ranging (LiDAR) data, available for building extraction. Generally, a photogrammetric image has three colour channels and may have an additional infrared channel, whereas LiDAR data mainly contain height information. Both sources have their own limitations such as photogrammetry imagery does not work well in certain weather conditions, whereas the LiDAR data have low point-density that causes small buildings to be undetected in dense vegetation regions. Both data sources require large number of manual settings to distinguish buildings from trees. In addition, the LiDAR data are prone to height error when representing transparent building roofs as the LiDAR points could be reflected from different levels of transparent roofs and objects under the transparent roofs.

Based on the input data to the building extraction methods, Lee et al. [1] categorised the building extraction methods into three different classes. The first class contains methods that use 2D or 3D information from photogrammetric imagery to extract building [2]. However, the 2D derived information, e.g., edges and textures, is inadequate to extract building completely and misclassification is very common, especially in very high resolution images. This problem is further compounded by the presence of shadows cast on the objects [3]. The 3D information, e.g., depth derived by multiple images, is inaccurate due to the mismatch between different capturing view angles [4].

The second class of methods uses LiDAR data only. Compared with photogrammetry imagery, LiDAR data provide more accurate height information [5,6,7]. By applying a height threshold, the LiDAR data are classified into ground and non-ground groups. Next, the ground LiDAR points are used to generate the building mask [8]. However, the transparent buildings contain ground points along with non-ground point, and therefore, these buildings are easily missed in building mask process [9]. This class also includes methods which use the filters such as recursive terrain fragmentation filter and morphological filter in order to group LiDAR data into off-terrain and on-terrain points [10,11]. However, the low LiDAR point density affects the building extraction accuracy of these methods. Alternatively, the LiDAR points are interpolated to preserve the sharp edges of buildings [12,13], but the manually generated LiDAR points might lead to false building extraction.

In the third class, instead of using data from only one system, the integration of the LiDAR and photogrammetric imagery data provides much better results in building extraction [5,7,14,15]. This is because each of these two types of data contains useful information about the terrain and the objects that the other type lacks. Different information derived from the photogrammetric imagery is merged with LiDAR height information, for example, some methods merge the Normalized Difference Vegetation Index (NDVI) with LiDAR height information to extract buildings [16,17], whereas other methods merge various types of information, e.g., textures, NDVI and edges, derived from photogrammetric imagery with LiDAR height information to extract buildings [5,7]. However, the methods in the third class still have limitations, e.g., (1) limited robustness in extracting buildings with fully or partially transparent roof as well as buildings which are partially occluded or small [7,9], and (2) not fully automatic as parameters are empirically tuned based on the set of the training areas [15] and therefore are ineffective in other environments.

This paper proposes a new Gradient-based Building Extraction (GBE) method (i.e., belongs to the third class), which is more robust in extracting buildings of a greater range of sizes, including the smaller buildings. In addition, the proposed GBE method can extract the transparent buildings as it does not use the building mask derived from the ground LiDAR points which would mark transparent portions of a building as ground objects. In the proposed method, the initial building cue, i.e., the prominent orientations of buildings, are first determined by analysing straight edges/lines extracted from the photogrammetric imagery. For each prominent orientation, a grid is set and the non-ground points are sampled based on the maximum height of the points within each cell in the grid. By calculating the maximum height of the LIDAR points, LiDAR points representing rooftop are extracted and this helps in extracting the transparent buildings. Next, the gradient is calculated in two orthogonal directions i.e., *X* and *Y* axes of the grid. The pixels whose gradient values are constant in direction are marked as pixels of building planes or regions. Instead of using a large area threshold to remove the small trees detected as buildings, the proposed method uses surrounding colour information of candidate building and matches it with that of the candidate. The candidate building is removed if more than 50% of its pixels are matched with the surrounding colour information. In cases where the candidate building is not matched up to predefined threshold, then only the matched pixels of the candidate building are removed. The LiDAR point variance and density analysis is also applied to remove vegetation. In addition, a shadow-based analysis is employed to eliminate the trees covered by the shadow of buildings.

The remainder of this paper is organised as follows: Section 2 provides an overview of the current state-of-the-art building extraction methods which are compatible to our proposed method. The limitations of these current methods and the main contributions of this research will also be presented in this section. Our proposed GBE method will be presented in Section 3. The experimental setup and the detail of the benchmark data sets will be described in Section 4. Section 5 presents the qualitative and quantitative results of the GBE method compared with four of the best current methods. Finally, Section 6 concludes the paper.

## 2. Related Work

In the past five years, the extraction of buildings from complex environments is trending towards using the combination of both photogrammetric imagery and LiDAR data. The methods using both types of data can generally be grouped into either the classification or rule-based segmentation approaches. Comparatively, the rule-based building extraction methods are more commonly used due to their simplicity and effectiveness for large range of environments. Our proposed method also belongs to this approach.

Generally, rule-based building extraction methods work as follows. First, the pre-processing stage provides the initial building cue. Second, this building cue is processed in main stage to extract the candidate building regions. Finally, the extracted building regions are verified in post-processing stage. Some methods only use the LiDAR data for searching of the initial building cue at pre-processing stage and it is further processed in the main process using the spectral features derived from the photogrammetric imagery [7,18]. In Cao et al. [19], the initial building cue is generated from the photogrammetric imagery using the Gabor and Morphological filters of a certain window size (a.k.a. structure element). Next, the building cue is further processed in the main process using the LiDAR data. The methods using the photogrammetric imagery and LiDAR data in every stage yield better results than the methods using either of them in each stage. Earlier, both types of data were only employed at the pre-processing stage to segment the sites into building and other groups [20], but later they are employed in every stage to extract buildings [21,22]. In Chen et al. [21], the region-based segmentation is employed on the photogrammetric imagery and LiDAR data to find the initial building cue. The initial building cue is then further processed using the rule-based segmentation to extract buildings. Sohn and Dowman [22] employed a classification algorithm to find the initial building cue from the photogrammetric imagery, LiDAR data and their derived data i.e., NDVI, Digital Surface Model (DSM) and Digital Terrain Model (DTM). The initial building cue is then further processed using the model-driven and data-driven approaches to extract rectilinear building edges. The trend of using derived data is followed by many methods [3,5,23,24,25]. In the post-processing stage, these methods employed the variance analysis and morphological filter to verify the buildings.

The derived information from photogrammetric imagery can be highly affected by the shadows casted on buildings [3]. Thus, some shadow indexes have been introduced to overcome this problem. These shadow indexes use the Hue, Saturation and Intensity (HSI) model where the saturation and intensity channels are used to detect the shadows [26]. In [26], the Ostu method is applied on the HSI derived indexes, i.e., difference index (*S* − *I*), ratio index (*S*/*I*) and normalization difference (*S* − *I*/*S* + *I*) where *S* is saturation and *I* is intensity, for detecting shadows. However, these indexes also incorrectly detect the dark blue and dark green regions of other non-shadow objects as shadows. This problem can be reduced by applying a threshold on the Ostu method [27], and by modifying the index formulae to measure Hue, Intensity (brightness) and Saturation [28]. Recently, three indexes, i.e., Morphological Shadow Index (MSI), Morphological Building Index (MBI) and Geometrical Index (GI) are proposed. These are collectively applied to eliminate the shadows and to extract buildings from the high resolution satellite imagery [29].

### 2.1. Limitations of Rule-Based Building Extraction Methods

In rule-based building extraction methods, LiDAR points with different heights on transparent buildings are used in building extraction process and as a result, (1) these LiDAR points are classified as tree points or (2) these points are classified as ground points and produce a ground region in a mask [9]. In addition, the transparent buildings have similar colour information of ground and thereby transparent buildings are undetected by applying image analysis as well.

**Figure 1:**
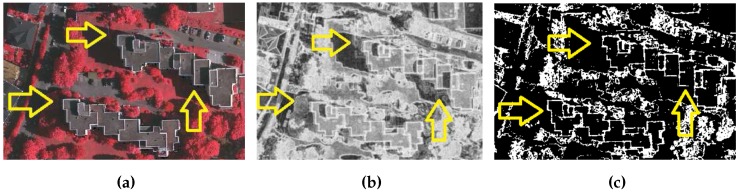
False building detection at yellow arrowed regions. (**a**) Photogrammetric Image; (**b**) Texture Index; (**c**) Texture mask.

Furthermore, the rule-based building extraction methods use empirically defined threshold of filter size to remove small regions as a remaining portions of trees. However, some sites contain small buildings and are removed by applying the large filter size threshold. In addition, colour derived indexes such as NDVI and texture are sensitive to shadow [5]. For example in Figure 1, the shadowed portions of trees, which are indicated by yellow arrows, have low texture index value and are extracted as building portions if a texture threshold is applied (e.g., 0.8 [5]). This example confirms the low discriminative power of the texture index.

**Figure 2:**
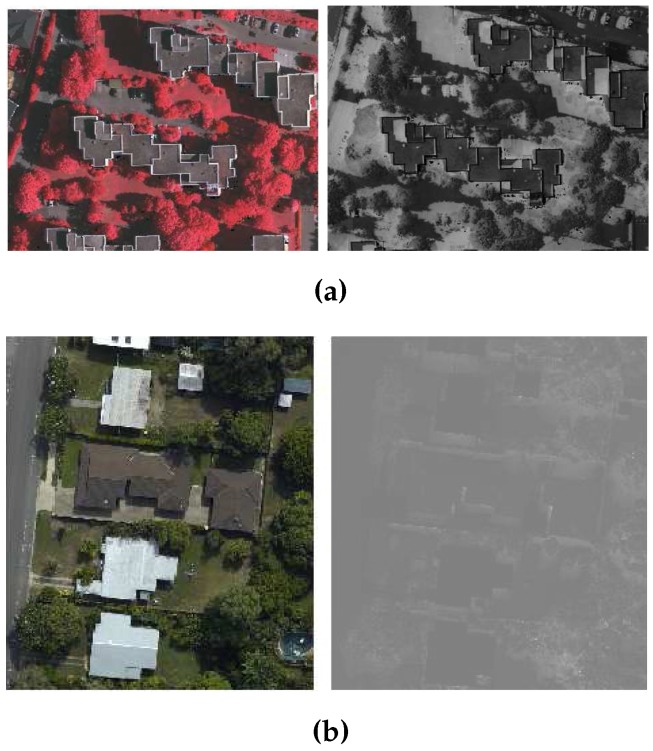
Different Pseudo-NDVI values for trees with (**a**) red; and (**b**) green leaves (To increase the readability of Figure 2b, brightness level of the Pseudo-NDVI image is increased by 90%. The original image is mainly black and when used as input into the proposed method, it has resulted in lower performance).

As for NDVI, it needs proper selection of colour channels of photogrammetric image [30]. For example, two channels, i.e., red and infrared channel, are selected manually to generate the NDVI index that can separate the green colour trees from buildings. If the red colour trees are present in the site, then the red channel is replaced with the green channel to separate the red colour trees from buildings. The provided data sets, i.e., Australian and German data sets [31], do not have a infrared channel. Therefore, a pseudo-NDVI is used as an alternative index. It is calculated by following the process in [7,24], where the red, green, and blue (RGB) channels are interpreted as Infrared, Green and Blue (IGB) channels respectively. The pseudo-NDVI results show different index values for locations with red and green trees, and therefore, it is very hard to tune a NDVI threshold. This phenomena is also verified in Figure 2, where trees in red and green colour shown different NDVI index values with respect to the surrounding buildings. Like the thresholds of colour derived indexes, and size filter, many other parameters of rule-based building extraction methods are also empirically tuned based on the set of training sites. Therefore, the robustness of the rule-based building extraction methods is limited to the environments present in the training sites.

### 2.2. Contributions of the Research

In this paper, we propose a novel Gradient-based Building Extraction method which uses the photogrammetric imagery and LiDAR data to utilise the benefits of both data. This research addresses two main problems, i.e., misclassification of small and transparent buildings, and sensitivity of rule-based parameters. To address the first problem, we propose a new method which does not use ground LiDAR points in generating the building mask, and therefore, the non-ground points on the transparent buildings are further processed in building extraction. In addition, the proposed method reduces the misclassification of non-ground LiDAR points of different heights as rooftop non-ground LiDAR points. Therefore, the rooftop non-ground LiDAR points are used to represent buildings with transparent roof. As mentioned in the Section 2.1, the large number of empirically tuned parameters reduce the robustness of rule-based building extraction methods. We propose a novel local colour matching analysis and shadow elimination procedure, where parameters are automatically set based on the local information of a given data and based on general conditions, e.g., darker region is a shadow region. These analyses replace the NDVI and texture. As a result, the introduced concepts not only are more adaptive in eliminating the vegetation from the sites, but also help in reducing the number of empirically set parameters required by the proposed building extraction method.

## 3. The Proposed Gradient-Based Building Extraction Method

**Figure 3:**
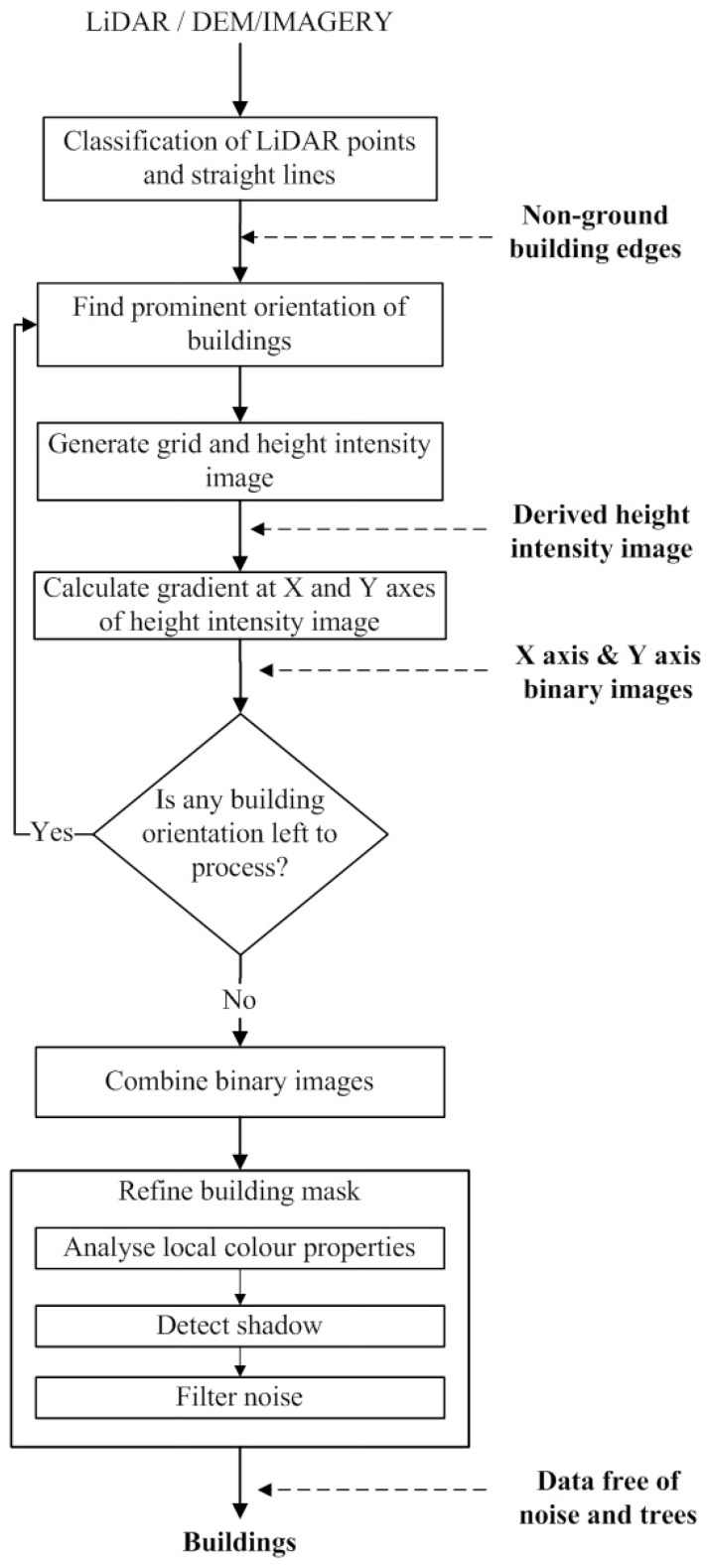
The flow diagram of the proposed method.

Figure 3 shows the flow diagram of the proposed GBE method. The inputs to the GBE method are the photogrammetric imagery and the LiDAR data of a given site. The LiDAR data are first divided into ground and non-ground points and then straight lines (i.e., principal orientations of buildings) are extracted from the photogrammetric imagery. The non-ground points help in separating non-ground lines from the ground lines. The principal orientations of buildings, i.e., initial building cue, are estimated using the non-ground lines. For each principal orientation *i*, the non-ground points are employed to generate an intensity image. The binary building mask $M_{i}$ in direction *i* is derived through a gradient analysis on the intensity image. Then, the masks $M_{i}$, 1≤i≤n, where *n* is the number of principal directions, are combined for a refinement process to remove trees. In the refinement process, the variance and density analysis is employed on the non-ground building points to eliminate trees, whereas, the local colour matching and shadow elimination analyses are employed on the imagery pixels to eliminate the remaining regions of trees. Some of the remaining tree pixels can be removed by using the morphological filter. Finally, the building boundaries are extracted around each building.

### 3.1. Classification of LiDAR Points and Straight Lines

**Figure 4:**
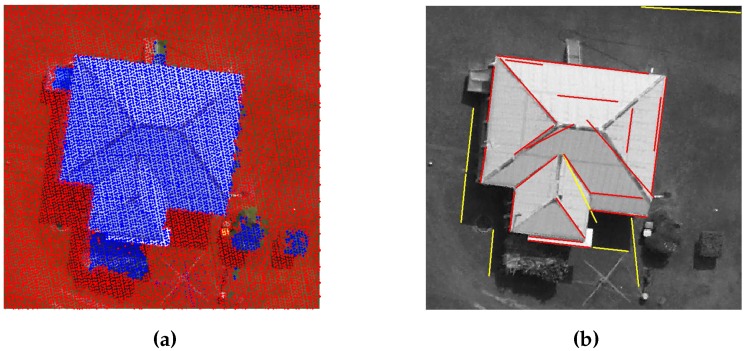
(**a**) Ground (red colour) and Non-ground LiDAR (blue colour) points (**b**) Building edges (red colour).

A DEM, i.e., bare earth height information, is derived from the LiDAR data with the help of a commercial software i.e., [32]. The bare-earth height information is subtracted from the LiDAR data to generate LiDAR points that are free of the terrain height. Later, the LiDAR points are grouped into ground and non-ground LiDAR points using a height threshold. An 1 m height threshold has been used to group the LiDAR points by many researchers [9,15,30]. Therefore, the 1 m height threshold is also used in the proposed GBE method to group the LiDAR points into ground and non-ground LiDAR points. Figure 4a shows the ground and non-ground points for a sample scene.

In addition, the straight lines are extracted from the photogrammetric imagery following the procedure in [5,33]. First, Canny Edge detector and Gaussian function are employed to extract straight lines from the photogrammetric imagery. Then, considering that buildings are at least 3 m in length [7], a 3 m length threshold is applied to extract the straight lines which are longer than 3 m. Finally, the non-ground LiDAR points are used to separate the non-ground straight lines from the ground straight lines. The non-ground straight lines represent buildings’ edges and ridges. The extracted straight lines from the sample scene are shown in Figure 4b, where that the ground lines and the non-ground lines are highlighted in yellow and red colours, respectively.

### 3.2. Finding Prominent Orientation of Buildings

**Figure 5:**
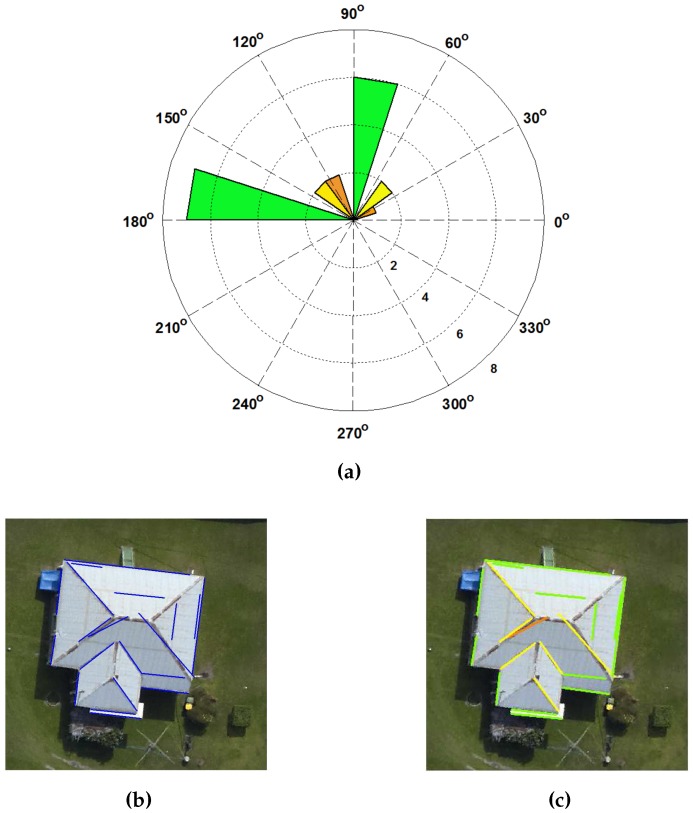
(**a**) An angular histogram of building edges, where the angular scale is defined in degree unit (**b**) Building edges and (**c**) Group of prominent building edges.

Finding prominent orientation of buildings is a complicated task. The extracted straight lines are used to estimate the prominent building orientations as follows. The angles of these lines are estimated between 0° and 180°. Then, these lines are grouped into 16 bins at $T_{dr}$ = 11.25° per bin size. This angle threshold is based on the assumption that buildings in a given scene are not randomly oriented. They are mainly oriented at around 0° (parallel), 90° (perpendicular), 45° (diagonally), 22.5° or 11.25° to one another. Then, mean angle *i* of each group is calculated to find the prominent direction of each group. Thus, approximately 16 prominent directions can be obtained and will be arranged in descending order according to size of the angle groups. Since, we are considering buildings of rectilinear shapes, each pair of bins which are perpendicular to each other will generate one prominent building orientation. For example, as shown in Figure 5a, there are pairs of orthogonal orientations.

We illustrate the calculation of the prominent building edges with an example shown in Figure 5b. There is a building which contains 20 edges at its building boundary and ridges. The threshold $T_{dr}$ is applied to divide the building edges into 16 groups based on the edge angle. An angular histogram of building edges, as shown in Figure 5a, is then generated. The three prominent building angles, *i* = [81°, 26° and 39°], are obtained. A green colour class, i.e., *i* = 81°, contains 13 building edges. The other seven building edges are grouped into yellow colour class (i.e., *i* = 26°) and orange colour class (i.e., *i* = 39°) respectively. The fourth quadrant of the angular histogram represents the perpendicular building edges of each group in a first quadrant. The building edges of each group are also plotted on an image, as shown in Figure 5c.

### 3.3. Generating Grid and Height Intensity Image

**Figure 6:**
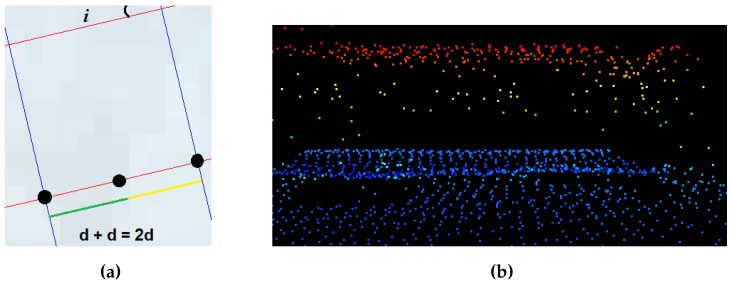
(**a**) Grid cell properties, and (**b**) LiDAR points on transparent building.

**Figure 7:**
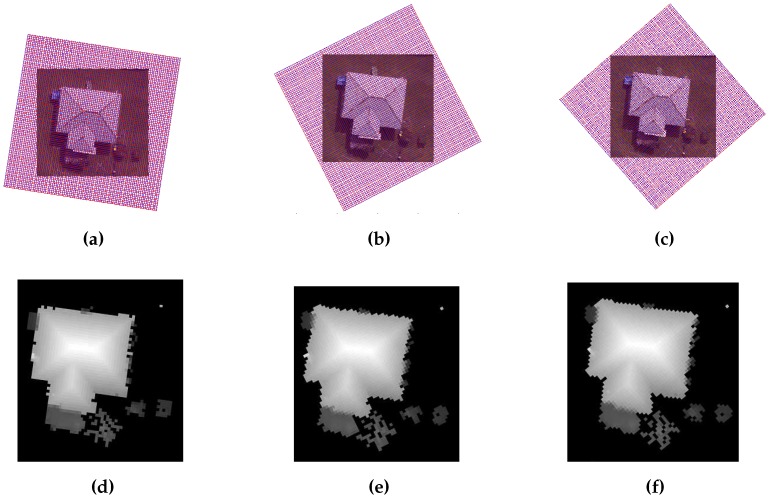
Grid overlaid on an image at (**a**) 81°; (**b**) 26°; and (**c**) 39°; Derived intensity image from LiDAR data at (**d**) 81°; (**e**) 26°; and (**f**) 39°.

For each estimated principal orientation, a height intensity image is created using a prominent orientation of building and a grid of resolution Td = 2*d*, where *d* represents maximum point-to-point distance in the LiDAR data. If the value of *d* is not available, it can be estimated using input point-density. Figure 6a shows the cell size and the direction of a grid. Then the conversion of 3D LiDAR information into intensity image is performed and *m*, i.e., the maximum of the LiDAR height values in the cell, is assigned as $P_{\left(j,k\right)}$, i.e., pixel of the height intensity image. Here, notation (j,k) is the coordinate of height intensity image. Thus, the height intensity images at three prominent directions of lines, as shown in Figure 7, are generated.

The advantage of using maximum is that it helps to reduce the height error of non-ground LiDAR point of transparent building, therefore, the transparent buildings can be extracted. Like maximum, mean can also be used to reduce the height error but mean cannot extract rooftop LiDAR points at multistorey transparent roof or transparent window buildings. The multistorey glass buildings would result in LiDAR points reflected from different floors close to transparent glass windows. The mean calculation extracts floor from the middle of the buildings as a roof. Therefore, the maximum of the LiDAR points in a cell is used to generate the gradient based height intensity image.

We illustrate the advantage of using maximum with an example shown in Figure 6b. There is a transparent building which contains LiDAR points at different heights. Therefore, if an incorrect LiDAR point is selected to represent a cell, then the transparent building will not be extracted. For example, Awrangjeb and Fraser [15] assigned the point closest to a cell center to cell. However, the center LiDAR point may not be reflected from rooftop of transparent building. Therefore, we assign the maximum of cell’s LiDAR points to cell that extract rooftop LiDAR point in a cell and also reduces the height error of non-ground LiDAR points among the cells. As a result, the transparent building will be extracted.

### 3.4. Gradient Calculation

**Figure 8:**
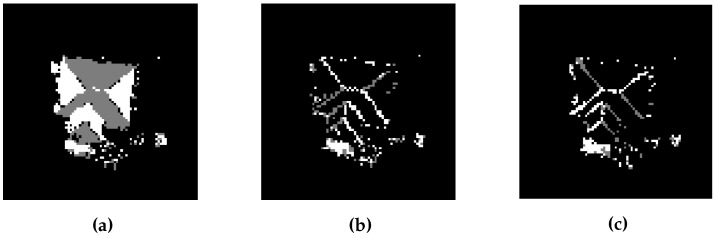
Gradient-based building mask at (**a**) 81°; (**b**) 26°; and (**c**) 39°; where constant gradient in *X* and *Y* axes are represent by white and gray colour.

Gradient is calculated using a differential function, which measures the slope magnitude in a direction of the greatest change. In the proposed method, gradient represents the slope and tangent of intensity changes among the pixels in each grid cell. The rate of change in intensity of a grid is measured in both *X* and *Y* directions of a grid. The building roofs generally have a constant rate of change in the intensity image, whereas trees have a random rate of change in intensity. A height tolerance threshold of 0.2 m is exploited to extract buildings. The regions with a rate of change in intensity less than 0.2 m are detected as building planes and are marked as building regions in gradient-based building mask. The constant rate of change in intensity on building planes is only observed when the grid is in the direction of building, otherwise a random rate of change in intensity on building planes is obtained. This phenomenon is also observed in Figure 8. Thus, the gradient calculation is vital to extract the transparent or opaque buildings of various sizes. There is a possibility that a site has multiple buildings at different orientations, and therefore, masks at every orientation are added to extract all buildings in a site.

### 3.5. Refine the Building Mask Using LiDAR Points

The gradient-based building mask may contain tree regions if dense trees, especially with flat tops, are present in a given area. In the proposed GBE method, the variance and density analysis [30] is used to remove the dense tree regions. The variance analysis is adopted based on the principle that LiDAR points representing trees have relatively higher variance as compared to those representing building roofs [34]. In order to calculate the variance, a window that is shifted over the non-ground LiDAR points of the gradient-based mask. In our method, the window size is equal to the twice of LiDAR point spacing Td, thereby, a window will contain at least one LiDAR point. A threshold of 0.2 m variance is set to eliminate the tree LiDAR points from non-ground LiDAR points.

**Figure 9:**
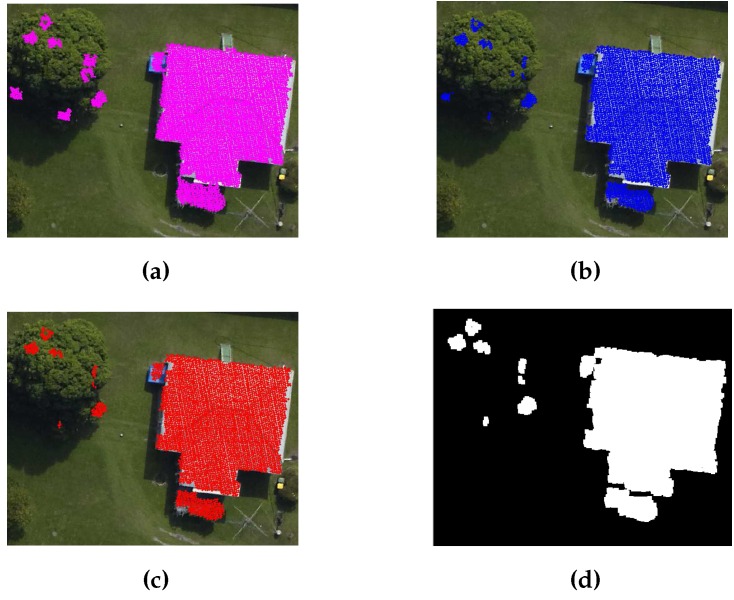
Results after applying variance and point density analyses: (**a**) LiDAR points at objects; (**b**) Removing the LiDAR points with high variance; (**c**) Examining the point density; and (**d**) Update the gradient-based building mask to finalize the result.

After applying the variance analysis, the density of the non-ground LiDAR points on tree is reduced as compared to building roofs which is also shown in Figure 9b. Therefore a window, i.e., cell size window, is shifted over the non-ground LiDAR points to analyse the LiDAR points density. The non-ground LiDAR points in a window are removed if they are less than half of the possible non-ground LiDAR points in a window. For example, if the non-ground LiDAR points density is 4 points/$m^{2}$ after variance analysis and is less than half of the actual non-ground LiDAR points i.e., 10 LiDAR points/$m^{2}$, then these LiDAR points will be removed. In Figure 9c, the LiDAR points of considerably high LiDAR point density regions are assigned as non-ground points of buildings. Finally, the remaining non-ground LiDAR points of buildings are used to update the gradient-based building mask. By using the density analysis followed by variance analysis, the LiDAR points on trees are either removed or reduced.

### 3.6. Refining Building Using Imagery

Dense vegetation may not be eliminated solely using the LiDAR data. Therefore, some existing methods [5,7] use additional information from the photogrammetric imagery, e.g., NDVI and texture, to eliminate vegetation. However, manual settings are required to derive such information from the photogrammetric imagery. For example, two channels, i.e., red and infrared, are selected manually to generate the NDVI data that can separate the green colour trees from buildings. If the infrared channel is absence, then a NDVI is estimated from the R, G and B channels. If the red colour trees are present in the site, then the red channel is replaced with the green channel to separate the red colour trees from buildings. Similarly, a window size has to be known beforehand to produce the texture information. In addition, thresholds are used in the NDVI and texture analysis to separate the trees from buildings. These thresholds are empirically tuned based on the characteristics of the training sets and their effectiveness to a given input may not be guaranteed.

The proposed method adopts a local adaptive approach, i.e., local colour matching analysis, to eliminate trees. This approach matches colour properties of objects with their surroundings and eliminates the matched objects. The unmatched objects are further processed by the shadow analysis. If objects are covered by shadow of buildings and have no straight lines, then those objects are also removed. Finally, a morphological filter is used to remove noise.

#### 3.6.1. Local Colour Matching Analysis

**Figure 10:**
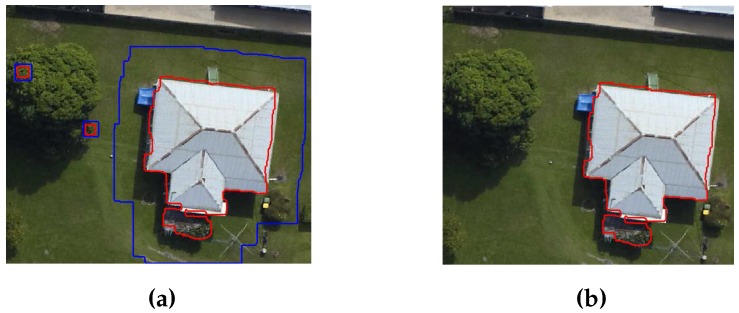
(**a**) Regions for local colour matching analysis, where the buildings are indicated by red boundaries and their surrounding regions are indicated by blue boundaries; and (**b**) Unmatched building region.

As mentioned earlier, a local colour matching analysis matches the buildings with their surroundings using colour information from the photogrammetric image. In terms of definition, the updated gradient-based building mask GM contains *B* number of buildings which are matched with the objects present in their surrounding area *A*. The size of area *A* around the building is equal to half of the size of that building. It is also shown in Figure 10a, where the building regions inside the blue boundaries are matched with their surrounded region (i.e., area *A* between the red and blue boundaries). The mean $T_{tc}$ of colour information of objects’ pixels present in the surrounding area *A* is measured. The extracted tree pixels from the gradient process are used to set a colour range threshold $T_{cr}$. The colour range threshold is defined as:

(1) $$T_{cr}=\left\{\begin{array}{c} min(r),max(r)\\ min(g),max(g)\\ min(b),max(b) \end{array}\right.$$

where the r, g and b are red, green and blue colour channels, respectively. Later, the number of matched pixels of the building are calculated by:

(2) $$BP=\left\{\begin{array}{c} r-\left(T_{tc}+T_{cr}\right)<BP_{r}<r+\left(T_{tc}+T_{cr}\right)\\ g-\left(T_{tc}+T_{cr}\right)<BP_{g}<g+\left(T_{tc}+T_{cr}\right)\\ b-\left(T_{tc}+T_{cr}\right)<BP_{b}<b+\left(T_{tc}+T_{cr}\right) \end{array}\right.$$

Finally, the building is removed if the value of BP is more than half of that building pixels. In other words, the buildings are removed if more than half of their pixels matched with $T_{tc}$. Later, the matched pixels of buildings are also removed to eliminate the trees that are connected to buildings. Using the building pixels, the building boundaries are drawn on the photogrammetric image. Generally, the implementation of the proposed local colour matching analysis is as follows:

1. Input the photogrammetric image and updated gradient-based building mask GM.
2. Extract *B* buildings from the GM.
3. Extract area *A* around the each of the *B* buildings.
4. Calculate $T_{tc}$ the mean of colour information in area *A* for building *i*, where *i*∈*B*.
5. Calculate $T_{cr}$ the colour range threshold using Equation (1).
6. Calculate the number of matched Pixels of the building BP according to Equation (2).
7. Eliminate the building if more than half of its pixels match with the $T_{tc}$.
8. Eliminate the matched pixels of building as well.
9. Repeat Steps 4 to 8 until all the buildings are processed.

#### 3.6.2. Shadow Analysis

**Figure 11:**
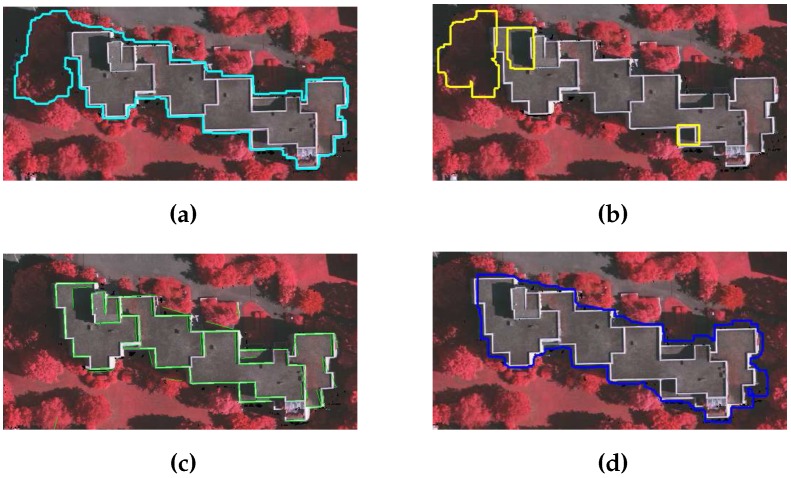
(**a**) Building region after applying the colour matching analysis; (**b**) Shadow region outlined in yellow colour; (**c**) Green straight lines around the building and (**d**) Building region in blue colour is extracted after applying the shadow analysis.

Dense trees covered by the shadows of buildings are not eliminated by the local colour matching analysis. This is also shown in Figure 11a, where the shadowed region is extracted as a part of the building. Therefore, a two-step shadow analysis is also defined in our proposed method as follows:

1. **Step 1 (Detect shadow region):** The HSI image of the input photogrammetric image is derived, where *I* is intensity and its range is [0, 1]. The lower value of *I*, e.g., $I_{s}$ = 0.25, indicates shadow region. Therefore, pixels having *I* less than $I_{s}$ are grouped as shadow regions which are also shown in Figure 11b.
2. **Step 2 (Eliminate shadow region):** The shorter trees and buildings may be covered by the shadows of the taller buildings. Interestingly, the shadowed buildings may have some long straight lines (i.e., extracted during initial steps by Canny edge detector), whereas shadowed trees have no long straight lines. This is also shown in Figure 11c, where the shadowed buildings have some straight lines and shadowed trees have no straight lines. Therefore, the straight lines are used to distinguish the shadowed trees from shadowed building. The shadowed tree pixels are eliminated and the building pixels are used to update the building regions. Finally, the building regions are masked into a refined mask.

#### 3.6.3. Morphological Filter

Morphological filter uses non-linear operations, e.g., opening function or dilation followed by erosion, to eliminate objects smaller than a predefined structure element (SE). The refined mask still has some smaller portion of trees. Therefore, the SE of size 1 $m^{2}$ is used to eliminate all objects below the 1 $m^{2}$. The refined mask, i.e., the updated gradient-based building mask, contains only the buildings. Using the refined mask, the buildings boundaries are outlined on a given photogrammetric imagery.

## 4. Experimental Setup

This section describes the parameter settings of the proposed GBE method. In addition, the data sets and the evaluating systems we have used for studying the performance of the proposed GBE method are also described in detail.

### 4.1. Parameter Settings

Table 1 shows the list of parameters used by the proposed GBE methods. Many of these parameters are adopted from the existing building extraction methods. Two empirically set parameters, i.e., height and straight line length thresholds, are used by the proposed GBE method to extract initial building cue from the photogrammetric and LiDAR data. These two parameters were previously used by Awrangjeb et al. [5,15,33]. The gradient process of the proposed GBE method uses three set parameters i.e., height intensity tolerance, cell length and degree range thresholds. The cell length and degree range thresholds are the same as those used in literature [5,15,33]. The cell length is set based on the LiDAR data resolution and the degree range threshold is empirically set. The height tolerance threshold of 0.1 m was proposed by Sampath and Shan [35]. However, this height tolerance threshold was not tested on the low resolution ISPRS data set [35]. In this research, the height tolerance value is set at 0.2 m, thereby the proposed method becomes more robust for low resolution data as well.

The refinement process of the proposed GBE method uses a total of six thresholds. These parameters are set using the photogrammetric image and LiDAR data. From the LiDAR data, variance and point density thresholds are set empirically to eliminate trees. The variance threshold value is defined using the value of height tolerance threshold (i.e., defined in gradient calculation). Siddiqui et al. [30] set the point density threshold value and the same value is used in this paper. Two more thresholds, i.e., the colour range and tree colour thresholds, are set automatically based on the photogrammetric image. The other thresholds, i.e., the building portion matched and shadow intensity thresholds, are set based on our following general observations on the relationship between buildings and their surrounding objects on the image. If the 50% of object is matched with its surroundings, then object is surely a building object. Therefore, the building portion matched threshold is set at 50%. As mentioned in the Section 3.6.2, the lower intensity value regions are most probably the shadow regions and this is also confirmed by analysing the straight lines at shadow regions. Therefore, the shadow intensity threshold is set at a lower value i.e., 0.25.

In addition, it is important to highlight that the proposed GBE method uses fewer parameters as compared to the existing methods. For example, Awrangjeb and Fraser [15] used 20 empirically set parameters for building extraction.

### 4.2. Benchmark Data Sets

**Figure 12:**
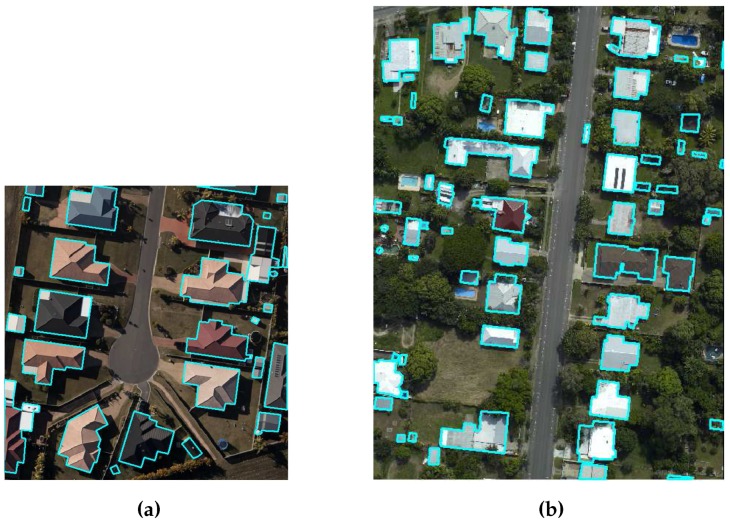
CRCSI benchmark data set i.e., (**a**) Harvey Bay and (**b**) Atkinvale, where the reference buildings are indicated by cyan boundaries.

Two benchmark data sets, i.e., the CRCSI data set [15] and ISPRS data set [36], are used to study the performance of the proposed GBE method in extracting buildings. The CRCSI data set contains two areas, Harvey Bay and Atkinvale of Australia. Both areas are depicted in Figure 12. Harvey Bay (HB) covers an area of 108 m × 104 m and has a point density of 12 points/$m^{2}$. It contains 26 single-storey residential buildings of different sizes that are surrounded by trees cut in different shapes. In addition, three buildings in this area have transparent roofs. Atkinvale (AV) covers an area of 214 m × 159 m and has a point density of 29 points/$m^{2}$. It contains open field with different objects including cars, trees, road and 63 buildings of different sizes. For example, four buildings are between 4 and 5 $m^{2}$, 10 buildings are between 5 and 10 $m^{2}$ and the rest are bigger than 10 $m^{2}$. This area also has a building with transparent roof. In terms of topography, HB is hilly and AV is flat. For the Australian sites, DTMs with 1 m resolution are available [15].

**Figure 13:**
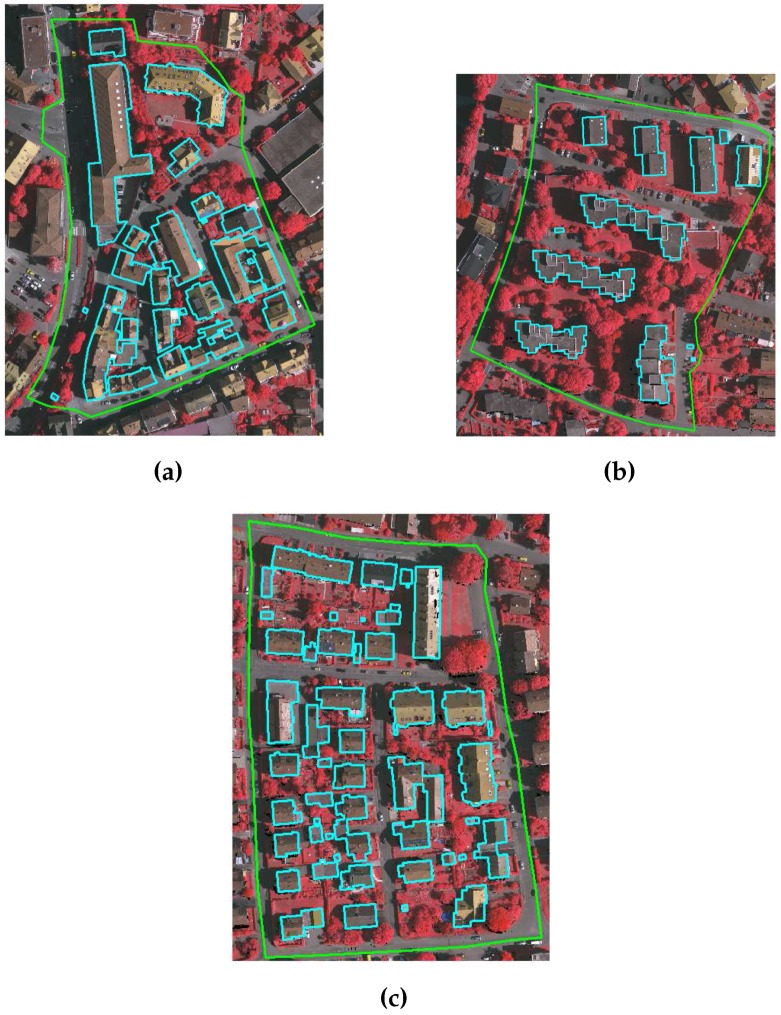
ISPRS benchmark data set (**a**) VA01; (**b**) VA02; and (**c**) VA03; where the benchmark areas are indicated by green boundaries and reference buildings are indicated by cyan boundaries.

The ISPRS data set contains dense vegetation and hilly areas of Vaihingen town of Germany. This data set consists of three areas as indicated by the green boundaries in Figure 13. The key characteristics of each area of the German site are as follows: Area VA01 is situated in the centre of the city of Vaihingen. It is characterised by dense development consisting of multistorey residential buildings having rather complex roof shapes such as gable and hip roof with small chimneys and dormers. In addition, the buildings are located near to trees with red leaves and some of the buildings are partially covered by trees. Area VA02 is characterised by multistorey residential flat roof buildings that are surrounded by dense vegetation, whereas Area VA03 is a residential area with small detached single-storey houses that are surrounded by dense vegetation. The point density of three areas of Germany is approximately 4 points/$m^{2}$, and the number of buildings (larger than 2.5 $m^{2}$) in VA01, VA02 and VA03 areas are 37, 14 and 56 respectively.

### 4.3. Evaluation Systems

To evaluate the performance of our proposed GBE method, the publicly available reference benchmark of German data set is used [31], whereas the reference benchmark for Australian data set is obtained using the BARISTA [37]. In addition, the ISPRS adopted threshold-based evaluation system [38] and the threshold-free evaluation system [39] are employed to evaluate the performance. Data collectors of both benchmark data sets define their own evaluating system to evaluate the performance of building extraction methods on their data sets. Therefore, we are also using the same evaluation systems defined for each of these data sets to compare the performance of the proposed method with the existing methods.

Three categories of evaluation i.e., object-based, pixel-based and geometric are used in both evaluation systems [38,39]. Each category of evaluation includes a few metrics to evaluate performance of the proposed method. The object-based metrics (i.e., completeness ($C_{m}$), correctness ($C_{r}$), quality ($Q_{l}$), over-segmentation (1:M), under-segmentation (N:1) and over-and-under segmentation (N:M) errors) estimate performance by counting the number of building objects, whereas the pixel-based metrics (i.e., completeness ($C_{mp}$), correctness ($C_{rp}$) and quality ($Q_{lp}$)) estimate the performance by counting the number of pixels of the extracted objects. In addition, the geometric metric i.e., Root Mean Square Error (RMSE) indicates the accuracy of the extracted building boundaries with respect to the reference building boundaries. The complete description of the above metrics are defined in [38,39]. The minimum areas of the large buildings have been set at 50 $m^{2}$ and 10 $m^{2}$ in the ISPRS benchmark and in the CRCSI benchmark, respectively. Therefore, the metrics i.e., completeness, correctness and quality, are separately measured for different sizes of buildings.

## 5. Experimental Results and Discussion

The results and discussion on the ISPRS and CRCSI data sets are presented in the Section 5.1, whereas the comparisons of the proposed GBE method with existing methods are discussed in Section 5.2. In addition, the stability of existing thresholds and the proposed thresholds for refinement process are tested where the quality metric is measured at different values of thresholds.

### 5.1. Performance Analysis of the Proposed Method

**Figure 14:**
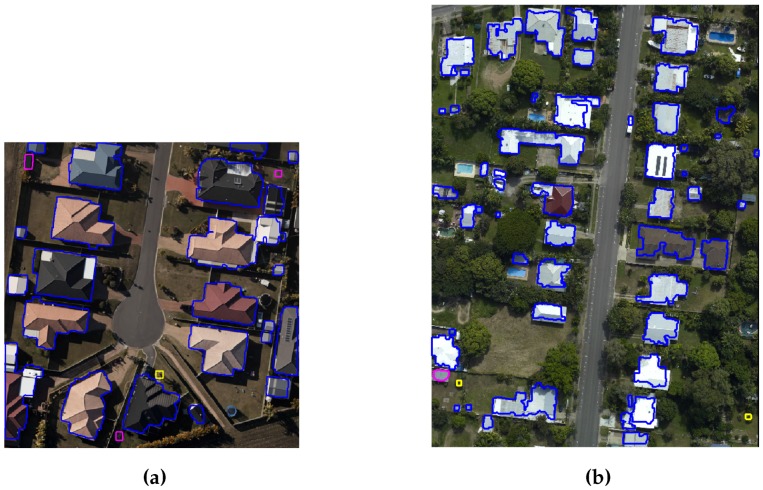
Qualitative analysis of (**a**) Harvey Bay(HB) and (**b**) Atkinvale(AV), where the buildings are indicated by blue boundaries, transparent buildings are indicated by magenta boundaries, and false detected buildings are indicated by yellow boundaries.

CRCSI data set: Quantitative and qualitative analyses for two areas, i.e., Harvey Bay and Atkinvale, of the CRCSI data set are performed following the procedure in [39]. Table 2 and Table 3 show the object and pixel based quantitative results, whereas the qualitative results are depicted in Figure 14, where the buildings are indicated by blue boundaries, transparent buildings are indicated by magenta boundaries, and false detected buildings are indicated by yellow boundaries.

The proposed GBE method extracts 25 buildings (i.e., highlighted by blue boundaries) from 26 reference buildings of HB area. In addition, the proposed GBE method extracts all three transparent buildings which are indicated by magenta boundaries in Figure 14. The proposed GBE method does not use ground points while creating the building mask where the transparent buildings were masked as a ground object [9]. Therefore, the GBE method is capable to extract transparent buildings, which are marked by magenta colour in Figure 14. There is only one undetected building which has many small size roof planes. These planes produce high variance in a small area and are eliminated during the variance and point density analysis. Similarly, the proposed GBE method extracts 55 buildings from 65 reference buildings of AV area. This is also shown in Figure 14b, where the GBE method successfully extracts all range of buildings (i.e., highlighted by blue boundaries) including a transparent building (i.e., highlighted in magenta colour). Due to the absence of objects in the surrounding area of trees, these trees have been detected as buildings by the matching analysis. In addition, if these trees are cut in to certain regular shapes, then these trees are not eliminated by the variance and density analysis. This phenomena is also shown in Figure 14b, where two trees (highlighted by yellow boundaries) are detected as buildings. In addition, many buildings are partially occluded by surrounding trees and have not been detected by the proposed GBE method. The object-based results are tabulated in Table 2, where the high average values of object and pixel-based correctness, completeness and quality indicate that the proposed GBE method is capable of extracting all range of buildings including the transparent buildings. In addition, the average values of object-based completeness, correctness and quality are all 100% for the buildings larger than 50 $m^{2}$, whereas the average value of object-based completeness, correctness and quality are all 93% for the buildings larger than 10 $m^{2}$. Similarly, the pixel-based results are tabulated in Table 3 and the average value of pixel-based correctness, completeness and quality are 87.4%, 92.7% and 81.3%, respectively.

**Figure 15:**
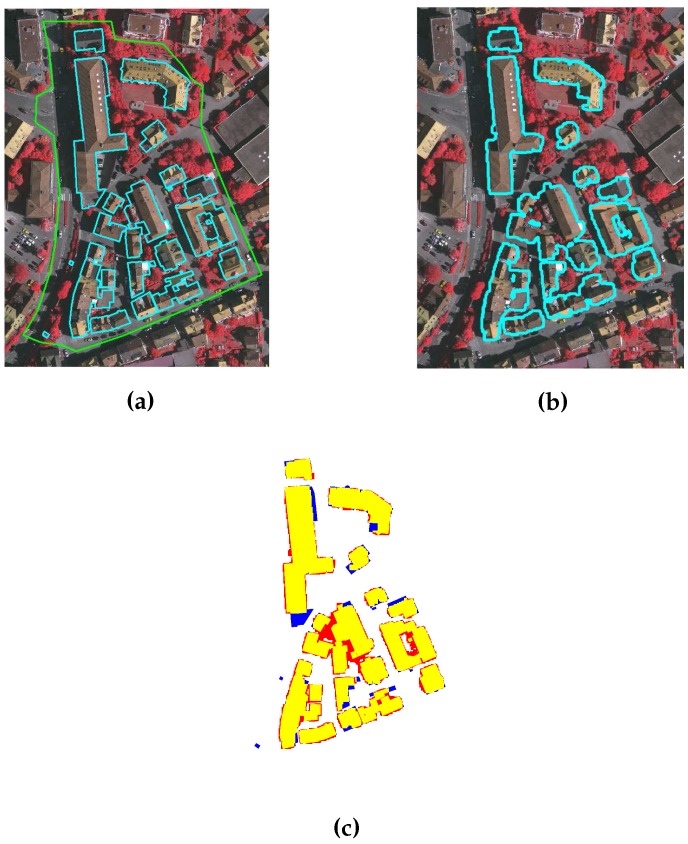
(**a**) Benchmark of VA01; (**b**) Building extraction after applying GBE; and (**c**) Result description by ISPRS, where pixels in yellow, blue and red colours are the true building extraction, missed building and false building extraction, respectively.

**Figure 16:**
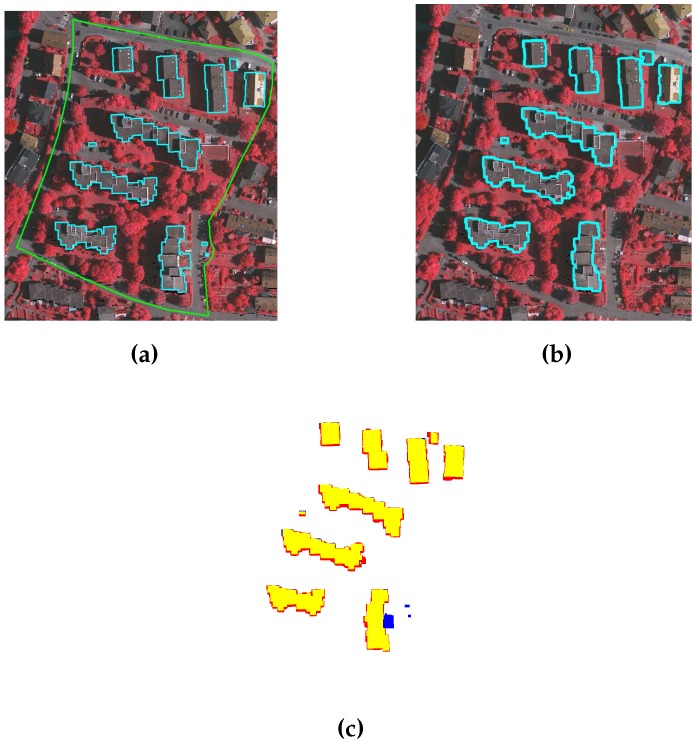
(**a**) Benchmark of VA02; (**b**) Building extraction after applying GBE; and (**c**) Result description by ISPRS, where pixels in yellow, blue and red colours are the true building extraction, missed building and false building extraction, respectively.

**Figure 17:**
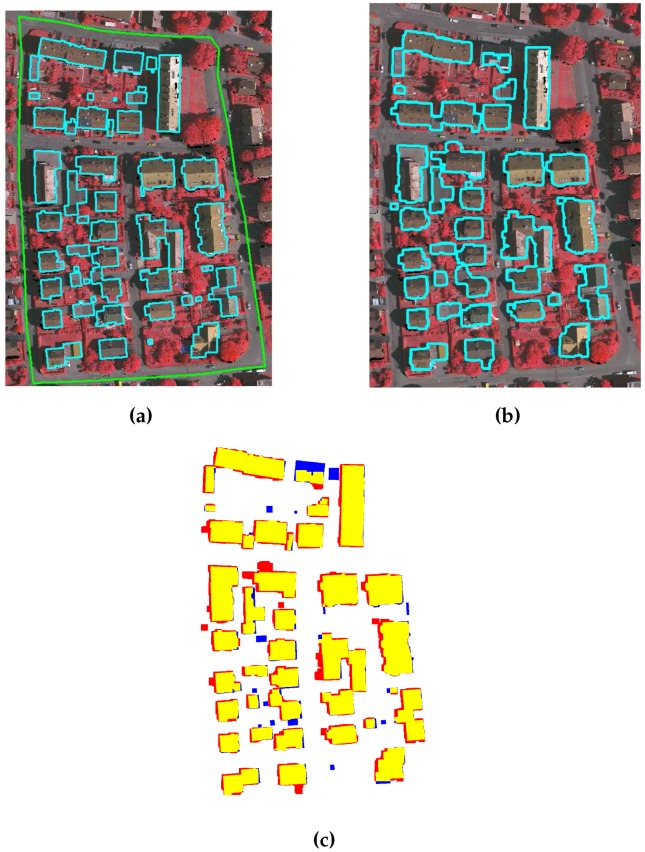
(**a**) Benchmark of VA03; (**b**) Building extraction after applying GBE; and (**c**) Result description by ISPRS, where pixels in yellow, blue and red colours are the true building extraction, missed building and false building extraction, respectively.

ISPRS data set: Quantitative and qualitative analyses for the three areas of ISPRS data set, i.e., VA01, VA02, and VA03, are performed following the procedure in [31]. Table 4 and Table 5 tabulate the object and pixel based quantitative results, whereas the qualitative results are depicted in Figure 15, Figure 16 and Figure 17. The results of the proposed GBE method for ISPRS data set are also available at the ISPRS portal with acronym MON3.

As shown in Figure 15, Figure 16 and Figure 17, the proposed GBE method extracts all the buildings which are taller than 1 m and larger than 1 $m^{2}$ size. In Figure 17, the building pixels are marked by yellow, whereas the pixels of undetected buildings are marked by blue. There are some false detected building pixels that are marked by red. From Table 4 and Table 5, the object and pixel based completeness, correctness and quality results also confirm that the proposed GBE method extracts all buildings which are larger than 1 $m^{2}$ and taller than 1 m. In addition, the buildings covered by trees are matched with surrounding trees and will be removed. In some cases, buildings are not extracted due to absence of the LiDAR data e.g., a large portion of buildings in top-middle of VA03 have no LiDAR data. Therefore, the object-based completeness and quality values are lower than 100% for buildings larger than 50 $m^{2}$. Under-segmentation is also present as shown by its non-zero value and this indicates that the two nearby buildings are detected as a single building. This occurred in the VA01 and VA03 areas, where the buildings are located very close to each other. In addition, the buildings in all three areas are not extracted multiple times, therefore, there is no over-segmentation for all three areas (please refer to Table 4).

### 5.2. Comparison Analysis

The proposed method does not use manually generated NDVI image. Therefore, the existing methods which do not use NDVI image are selected for comparison purpose. The list of compared methods are tabulated in Table 6. Using the CRCSI data set, the proposed GBE method is compared with a recently published method in [15]. The quantitative results of the compared methods using the CRCSI data set are tabulated in Table 7. The ISPRS data set is also used to study the performance of the proposed method as compared to MON2, KNTUmod, IIST2 and WHUQC methods. The first method, i.e., MON2, solely uses LiDAR data, whereas the other methods use both the photogrammetric imagery and LiDAR data. The quantitative results of the compared methods using the ISPRS data set are tabulated in Table 8.

In the CRCSI data set, the proposed GBE method obtains better results in both object and pixel based evaluation, except in AV in which the MON2 method produces better object and pixel based completeness. However, the object and pixel based correctness are better for the proposed GBE method. In addition, the object-based completeness and correctness for buildings larger than 10 $m^{2}$ are also better for the proposed GBE method.

Similar to the CRCSI data set, the proposed GBE method produces comparatively better results for the ISPRS data set. In Areas VA01 and VA02, the proposed GBE method produces better or comparable results in both object and pixel based evaluations, except the pixel based correctness where the WHUQC is better due to use of contour analysis (i.e., shape analysis). The contour analysis uses many empirically set parameters, thereby, the application of the WHUQC method is limited to the ISPRS data set. Nevertheless, the WHUQC method produces poor results in other metrics as compared to the proposed GBE method. In Area VA03, the proposed GBE method produces better results in pixel-based completeness and in object-based completeness and correctness for buildings larger than 50 $m^{2}$. The roofs in VA03 have colours similar to the trees and this does not allow the GBE method to extract them. Therefore, the GBE method produces poorer object-based results for VA03. In addition, some buildings of VA03 have no LiDAR points. By extracting buildings having no LiDAR points, the KNTUmod and WHUQC methods produce better results in pixel-based correctness. Both methods extract buildings based on an image only and LiDAR is employed for building refinement.

### 5.3. Stability Analysis

The stability of the proposed GBE method’s parameter, i.e., building portion matched threshold $T_{m}$, is evaluated by varying its value. Pixel-based quality is selected to measure the results at different parameter values. In order to compare the stability of proposed parameters, the two existing methods’ parameters i.e., used point ratio $u_{r}$ [9,15] and unused point ratio *u* [15] are selected. However, both existing methods have many other parameters which are not selected for stability test due to unavailability of their codes. For a fair comparison, the two existing methods’ parameters are applied on data that are generated by gradient process of the proposed GBE method.

After applying the different values of parameters on the VA02 and AV areas, the quality metric $Q_{l}$ is measured and tabulated in Table 9. In addition, the standard deviation is measured to explain the variation in quality at different values of parameters. Comparing the standard deviation results of existing methods’ parameters, the proposed parameter produces lowest standard deviation i.e., 0.004 and 0.215 in VA02 and AV areas respectively. This finding also confirms that GBE method is more stable as compared to the existing methods.

## 6. Conclusions

In this paper, a new gradient-based building extraction method is proposed. This gradient-based method is more robust in extracting of bigger range of sizes as well as transparent buildings. The proposed method uses LiDAR points in a direction of the extracted building lines from photogrammetric image to extract buildings. In addition, the local colour matching and shadow analysis are introduced, where some parameters are automatically set based on local information from a given image. This analysis replaces many empirically set parameters of the existing building extraction methods, and as a result, the proposed method uses fewer empirically set parameters. One of the introduced parameters, i.e., shadow threshold, is set based on a general observation, whereas a building match threshold is set empirically and it is more stable at different parameter values. The proposed method is compared with state-of-the-art methods for building extraction. Our experimental results show that our proposed method is more effective in extracting all kinds of building, i.e., small size buildings and buildings with transparent roof. Among the compared methods for ISPRS data set, KNTUmod is the only method which is consistent in generating the good results. Compared with the quantitative analysis of KNTUmod, the GBE method’s average completeness is 6.01% higher in pixel-based and 2.1% higher in object-based evaluation. The performance of our proposed method is further verified on CRCSI data sets and its results are compared with one of the most recent methods i.e., MON2. Compared with quantitative analysis of MON2, the GBE method’s average completeness is 12.9% higher in object-based evaluation and its average correctness is also 0.25% higher in pixel-based evaluation. Furthermore, our proposed parameters are more robust. Based on the stability test, the proposed parameters are comparatively more stable and have achieved the lowest standard deviation of quality i.e., 0.004 and 0.215 for VA02 and AV areas, respectively.

The proposed method mainly depends on LiDAR data to extract building planes. Therefore, the proposed method is unable to extract the planes which are smaller than the LiDAR point spacing. In addition, the proposed method is designed to extract buildings with flat and slope roofs. As a result, buildings with curved roofs, e.g., rainbow roofs, barrel roofs, bow roofs and dome roofs, will not be extracted by the proposed method. Our future work is to develop a more robust surface modelling procedure that can extract all kinds of curved roof.

## Figures and Tables

**Table 1:** Parameters used in proposed GBE method.

No.	Thresholds/Parameters	Values	Sources
1	Height $H_{t}$	1 m	[9,15]
2	Straight line length	3 m	[5,15,33]
3	Degree range $T_{dr}$	11.25	[15]
4	Grid/cell length	2 $d_{max}$	[15]
5	SE size	1 $m^{2}$	[15]
6	Height tolerance/Variance	0.2 m	this paper
7	point density in a grid	0.5	[30]
8	Building portion matched $T_{m}$	0.5	this paper
9	Colour range $T_{cr}$	automatically set	this paper
11	Tree colour $T_{tc}$	automatically set	this paper
12	Shadow intensity $I_{s}$	0.25	this paper

**Table 2:** Building extraction results: object-based evaluation for the CRCSI data set. $C_{m}$ = completeness, $C_{r}$ = correctness and $Q_{l}$ = quality ($C_{m,10}$, $C_{r,10}$, $Q_{l,10}$ and $C_{m,50}$, $C_{r,50}$, $Q_{l,50}$ are for buildings over 10 $m^{2}$ and 50 $m^{2}$ in percentage, respectively).

Areas	$C_{m}$	$C_{r}$	$Q_{l}$	$C_{m,10}$	$C_{r,10}$	$Q_{l,10}$	$C_{m,50}$	$C_{r,50}$	$Q_{l,50}$
**HB**	96.0	96.1	92.6	100	100	100	100	100	100
**AV**	76.9	96.3	75.7	86.2	100	86.8	100	100	100
**Average**	**86.4**	**96.2**	**84.1**	**93.1**	**100**	**93.4**	**100**	**100**	**100**

**Table 3:** Building extraction results: pixel-based and geometric evaluation for the CRCSI data set. $C_{mp}$ = completeness, $C_{rp}$ = correctness, $Q_{lp}$ = quality in percentage; RMSE = planimetric accuracy in metres.

Areas	$C_{\mathrm{mp}}$	$C_{\mathrm{rp}}$	$Q_{\mathrm{lp}}$	RMSE
**HB**	95.8	89.7	86.3	0.79
**AV**	79	95.7	76.4	1.1
**Average**	**87.4**	**92.7**	**81.3**	**0.94**

**Table 4:** Building extraction results: object-based evaluation for the ISPRS data set. $C_{m}$ = completeness, $C_{r}$ = correctness and $Q_{l}$ = quality ($C_{m,50}$, $C_{r,50}$, $Q_{l,50}$ are for buildings over 50 $m^{2}$) in percentage; 1:*M* = over-segmentation and *N*:1 = under-segmentation, *N*:*M* = both over- and under-segmentation in the number of buildings.

Areas	$C_{m}$	$C_{r}$	$Q_{l}$	$C_{m,50}$	$C_{r,50}$	$Q_{l,50}$	1:*M*	*N*:1	*N*:*M*
**VA01**	86.5	96.9	84.1	100	100	100	0	7	0
**VA02**	85.7	100	85.7	100	100	100	0	2	0
**VA03**	76.8	95.7	74.2	97.4	100	97.4	0	6	0
**Average**	**83.0**	**97.5**	**81.3**	**99.1**	**100**	**99.1**	**0**	**5**	**0**

**Table 5:** Building extraction results: Pixel-Based and geometric evaluation for the ISPRS data set. $C_{mp}$ = completeness, $C_{rp}$ = correctness, $Q_{lp}$ = quality in percentage; RMSE = planimetric accuracy in metres.

Areas	$C_{\mathrm{mp}}$	$C_{\mathrm{rp}}$	$Q_{\mathrm{lp}}$	RMSE
**VA01**	93.5	86.0	81.1	1.1
**VA02**	97.2	84.3	82.3	1.0
**VA03**	93.7	81.3	77.1	1.0
**Average**	**94.8**	**83.8**	**80.1**	**1.03**

**Table 6:** Existing methods used to compare with proposed method.

Methods	Published	Tested on Data Set
MON2	[15]	CRCSI and ISPRS
KNTUmod	[40]	ISPRS
IIST2	[41]	ISPRS
WHUQC	[42]	ISPRS

**Table 7:** Comparing building detection results for the CRCSI data set.Object-based $C_{m}$ = completeness and $C_{r}$ = correctness ($C_{m,10}$ and $C_{r,10}$ are for buildings over 10 $m^{2}$) and pixel-based $C_{mp}$ = completeness and $C_{rp}$ = correctness are in percentage; RMSE = planimetric accuracy in metres.

Areas	Methods	$C_{m}$	$C_{r}$	$C_{m,10}$	$C_{r,10}$	$C_{\mathrm{mp}}$	$C_{\mathrm{rp}}$	RMSE
**HB**	**GBE**	96.0	96.1	100	100	95.8	89.7	0.60
	**MON2**	80	95.2	90.9	95.2	91.3	90.0	0.84
**AV**	**GBE**	77.0	96.3	86.2	100	78.4	95.7	0.90
	**MON2**	67.2	100	81.1	100	87.2	94.9	0.66

**Table 8:** Comparing building detection results for the ISPRS data set. Object-based $C_{m}$ = completeness and $C_{r}$ = correctness ($C_{m,50}$ and $C_{r,50}$ are for buildings over 50 $m^{2}$) and pixel-based $C_{mp}$ = completeness and $C_{rp}$ = correctness are in percentage; RMSE = planimetric accuracy in metres.

Areas	Methods	$C_{m}$	$C_{r}$	$C_{m,50}$	$C_{r,50}$	$C_{\mathrm{mp}}$	$C_{\mathrm{rp}}$	RMSE
**VA01**	**GBE**	86.5	96.9	100	100	93.5	86.0	1.01
	**MON2**	83.8	96.9	100	100	92.7	88.7	1.11
	**KNTUmod**	78.6	100	100	100	91.4	94.3	0.8
	**IIST2**	83.8	84.8	89.3	96.3	90.7	82.4	1.2
	**WHUQC**	78.4	96.9	92.9	100	83.7	98.1	1.0
**VA02**	**GBE**	85.7	100	100	100	97.2	84.3	1.07
	**MON2**	85.7	84.6	100	100	91.5	91.0	0.8
	**KNTUmod**	78.6	100	100	100	86.5	93.6	1.0
	**IIST2**	71.4	91.7	100	90.9	86.2	90.3	0.8
	**WHUQC**	85.7	100	100	100	86.7	99.6	0.8
**VA03**	**GBE**	76.8	95.7	97.4	100	93.9	81.3	1.03
	**MON2**	78.6	97.8	97.4	100	93.9	86.3	0.89
	**KNTUmod**	85.7	98.0	100	100	88.3	99.0	0.7
	**IIST2**	83.9	53.2	94.7	82.2	91.0	75.7	1.1
	**WHUQC**	78.6	100	97.4	100	87.0	98.3	0.9

**Table 9:** Stability test of the proposed and existing parameters.

	GBE		MON2			
	$T_{m}$	$Q_{l}$	$u_{r}$	$Q_{l}$	***u***	$Q_{l}$
**VA02**	**40**	82.250	**40**	79.970	**0.45, 0.2**	80.633
	**50**	82.250	**50**	77.428	**0.55, 0.3**	80.268
	**60**	82.246	**60**	70.846	**0.4, 0.15**	79.391
	**70**	82.241	**70**	55.656	**0.35, 0.1**	79.527
	**St.D**	**0.004**	**St.D**	**10.912**	-	**0.594**
**AV**	**40**	76.699	**40**	68.751	**0.45, 0.2**	65.345
	**50**	76.407	**50**	71.186	**0.55, 0.3**	69.407
	**60**	76.474	**60**	64.197	**0.4, 0.15**	69.298
	**70**	76.878	**70**	47.740	**0.35, 0.1**	65.322
	**St.D**	**0.215**	**St.D**	**10.557**	-	**2.320**
